# Subclavian arterial rupture due to blunt trauma injury: A case report

**DOI:** 10.1097/MD.0000000000038775

**Published:** 2024-07-12

**Authors:** Suyeong Hwang, Gun Woo Kim, Sung Hoon Cho, Deokbi Hwang, Kyoung Hoon Lim

**Affiliations:** a Division of Trauma and Critical Care, Department of Surgery, Trauma Center, Kyungpook National University Hospital, School of Medicine, Kyungpook National University, Daegu, Republic of Korea; b Division of Vascular and Endovascular Surgery, Department of Surgery, Kyungpook National University, Daegu, Republic of Korea

**Keywords:** blunt trauma, endovascular approach, limb sacrifice, subclavian arterial injury

## Abstract

**Rationale:**

Subclavian arterial injury due to blunt trauma is rare but can have devastating outcomes. Massive hemorrhage or limb ischemia might develop depending on the extent of damage, and open repair might be necessary to salvage the limb. However, life-saving treatments should be prioritized in critically unstable patients.

**Patient concerns:**

A 21-year-old male patient who was transferred to our trauma center following a motorcycle accident. Abdominal and chest computed tomography (CT) revealed right renal injury and massive hemothorax with several rib fractures in the right chest.

**Diagnosis and interventions:**

Right renal injury with multiple extravasations and right 8^th^ intercostal arterial injury were detected during angiography. Emergent exploration with lateral thoracotomy was performed to manage right hemothorax. Pulsating bleeding from the thoracic roof observed in the operative field suggested a subclavian arterial injury. The unstable vital signs did not recover despite massive transfusion, and his right arm had already stiffened. Therefore, endovascular approach was adopted and the second portion of the right subclavian artery was embolized using microcoils and thrombin.

**Outcomes:**

Postoperative intensive care unit management performed to resuscitate patient from multiorgan failure included continuous renal replacement therapy (CRRT). After confirming the demarcation lines, transhumeral amputation of the right arm was performed on admission day 12. The patient recovered from multiorgan failure for more than 3 weeks after the accident; however, the patient survived.

**Lessons:**

Limb salvage, albeit critical for quality of life, is not possible in some cases where life-saving measures require its sacrifice. In these cases, quick decision-making by the surgeon is paramount for patient survival. As illustrated in this case, endovascular approaches should be considered less invasive measures to save the patient’s life.

## 1. Introduction

Subclavian arterial injury due to blunt trauma is a rare presentation, constituting <5% of all blunt trauma cases.^[1,2]^ Subclavian arterial injury is associated with high morbidity and mortality of approximately 24%.^[3]^ The estimated limb loss rate ranges from 2.4% to 2.9% in patients with subclavian or axillary arterial injury.^[4]^ Albeit relatively uncommon, these injuries pose an immediate risk to life due to exsanguination and diagnosis and treatment plan should be developed without delay. Here, we present the case of a patient with multiple injuries due to blunt trauma; the patient had to undergo limb amputation to preserve life. Ethical approval was waived by the medical ethics committee of Kyungpook National University Hospital to publish this study and the informed consents were given.

## 2. Case Presentation

A 21-year-old man without notable medical history was transferred to the Trauma Center of Kyungpook National University Hospital after a motorcycle accident. At arrival to the center, blood pressure and heart rate were 156/132 mm Hg and 108 beats/min, and hemoglobin level and platelet count were 13.3 g/dL and 240,000/μL, respectively. Abdominal contrast-enhanced computed tomography (CT) revealed grade III right renal laceration. Chest CT revealed significant hemothorax and several rib fractures in the right chest, requiring closed thoracostomy with a 28-Fr chest tube. The patient’s systolic blood pressure dropped below 60 mm Hg and follow-up evaluation revealed hemoglobin and platelet values of 7.5 g/dL and 170,000/μL, respectively. Renal injury and hemothorax were considered the main bleeding foci, and angioembolization was planned. Three pints of fresh-frozen plasma and 7 pints of red blood cells were administered in addition to norepinephrine until the procedure.

Right renal angiography revealed multiple extravasations flowing the ureter. The right renal artery underwent complete embolization using glue based on persistent substantial hematuria despite targeted embolization with coils and Gelfoam and overall parenchymal crushing injury.

Next, the right intercostal arteries at various levels were evaluated by angiography for treating significant hemothorax >1 L. Gelfoam embolization was performed for active extravasation in the 8th intercostal artery. However, bleeding persisted through the thoracostomy tube, and emergent surgical exploration was initiated by the thoracic surgeon.

**Figure 1.**
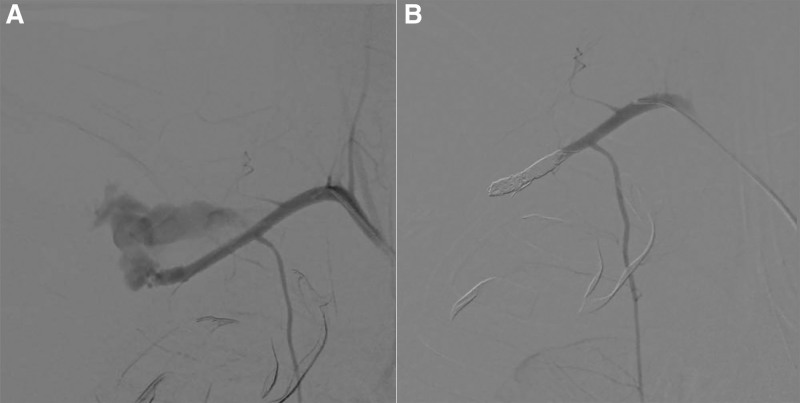
(A) Initial intraoperative angiography showing blushes of the distal subclavian artery. (B) Intraoperative angiography after coil embolization showing no contrast leakage.

Exploration during lateral thoracotomy revealed a fracture in the right first rib, but no injuries were observed in the superior vena cava or pericardium. In addition, pulsating bleeding from the thoracic roof was observed, raising the possibility of subclavian arterial injury. However, the patient’s vital signs remained unstable despite the transfusion of 15 pints of red blood cells and 10 pints of fresh-frozen plasma. Furthermore, the patient’s right arm had already stiffened. The above reasons made it difficult to explore or ligate the subclavian artery. Open repair would not aid in saving the limb; therefore, the endovascular route was adopted. Intraoperative angiography revealed distal rupture in the right subclavian artery, and advancing the guidewire to the true lumen of the distal artery failed (Fig. 1A). Therefore, the second portion of the right subclavian artery was embolized with several microcoils and thrombin (Fig. 1B).

Postoperatively, continuous renal replacement therapy (CRRT) was administered to correct oliguria and metabolic acidosis. On the 9 day of admission, an encapsulated epidural hemorrhage found in the C6–C7 level was removed with hemilaminectomy performed by a neurosurgeon. On the 12 day of admission, transhumeral amputation was performed after confirming the demarcation lines of the right arm. Hemodialysis was discontinued after the full recovery of renal function.

## 3. Discussion

Subclavian arterial injury due to blunt trauma is rare.^[5]^ The thoracic cage and clavicle, which provide adequate protection to the subclavian arteries underneath, also act as barriers for arterial access. Controlling hemorrhage from subclavian arterial injury requires median sternotomy or supraclavicular incision, both of which are technically challenging. These demanding maneuvers are even more difficult to perform in patients with blunt trauma due to the surrounding tissues and the rigid chest wall.^[6]^ Proximal and distal control of the injured vessel is critical in preventing massive blood loss; however, access and repair of the subclavian vessels are challenging due to their location.^[7]^ Therefore, open repair has its own morbidity and mortality.

Despite various efforts, in-hospital mortality rates reach 10% in patients with subclavian arterial injury, with 21% of deaths occurring due to hemorrhage.^[8,9]^ Although significantly improved outcomes in patients with subclavian arterial injury are observed, the primary goal of the treatment should be survival that requires prompt diagnosis. In addition to vascular signs such as the absence of peripheral arterial pulses and the presence of pulsatile bleeding, Sturm and Cicero reported various symptoms that should raise the suspicion of subclavian artery injury, including first rib fracture, brachial plexus palsy, pulsating supraclavicular hematoma, widened mediastinum, and localized hematoma over subclavian vessels.^[10]^ Unfortunately, only 20% of patients with multiple trauma exhibit vascular or hard signs,^[11]^ indicating that the diagnosis of subclavian arterial injury is difficult in these patients. Therefore, even in cases where the hard signs are overlooked, subclavian arterial injury should be strongly suspected if unexplained hypotension is accompanied by massive hemothorax or persistent bleeding from the thoracostomy tube.

**Figure 2.**
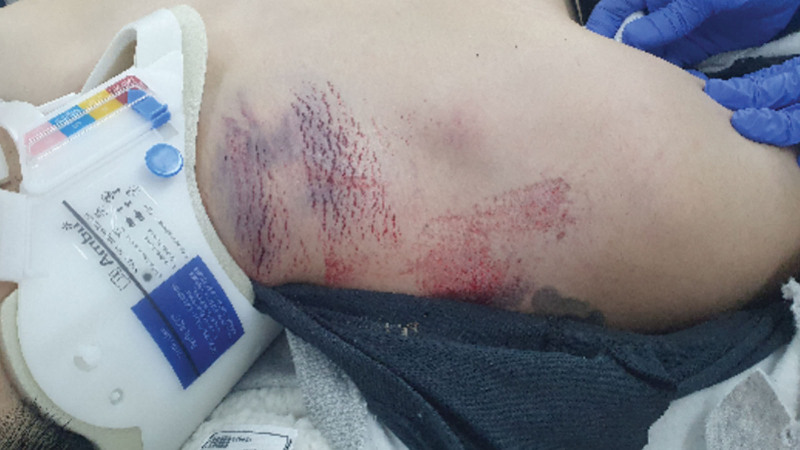
Bruise on the right shoulder at admission.

In patients with suspicious subclavian artery injury, contrast-enhanced CT is the most appropriate modality, followed by angiography to develop a precise treatment plan. Subclavian arterial injury is easily overlooked in the absence of strong suspicion. In this case, although a wound was found opening into the thoracic cavity in addition to hemothorax, external hematoma was not observed (Fig. 2).

Open repair of the subclavian artery is difficult to perform due to not only the anatomical location but also the wide dissection range, which is a serious concern in patients at high risk of disseminated intravascular coagulation. Endovascular therapy has become a preferred option for patients with subclavian arterial injury. Endovascular approaches require a smaller incision and are associated with fewer complications than open surgery. In 2017, the WTA (Western Trauma Association) group added management guidelines for subclavian and axillary artery injury after a multicenter review to the algorithm presented by Carrick et al in 2010.^[8,12]^ Accordingly, angiography can be used in patients who are hemodynamically stable with hard signs. However, open repair should be performed in unstable patients or those in whom access via catheterization fails. In the operating room, primary repair, or graft interposition can be considered through the open method.

Immediate surgery is recommended for hemodynamically unstable patients with hard signs.^[13,14]^ However, subclavian arterial injury inside the thoracic cavity requires attention to the surrounding tissue damage with clavicle dissection during open repair. This approach should be carefully considered in critically unstable patients, most of whom develop disseminated intravascular coagulation following massive hemorrhage.^[15]^ In such cases, life-saving damage control should be prioritized. Ligation via the open method is recommended^[16]^; however, endovascular approaches are less invasive methods than open repair in cases where a hybrid operating room or immediate intraoperative endovascular access is available.

This case illustrates that arterial embolization can be an effective alternative to ligation as an approach to control massive hemorrhage in hemodynamically unstable patients with subclavian arterial injury without increasing risk. As endovascular approaches become more popular, we suggest that this method may help improve the survival of patients with subclavian artery injury. Indeed, this was the best option, given the current patient’s unstable clinical condition. Recovery from multiorgan failure required 3 weeks after the surgery; however, the patient survived.

In summary, although critical for quality of life, salvage of a limb is impossible in some cases where life-saving measures require its sacrifice. In such cases, quick decision-making by the surgeon is paramount for patient survival.

## Acknowledgments

The authors would like to thank the patient and his family for the informed written consent for publication of this case report and accompanying image.

## Author contributions

**Writing – original draft:** Suyeong Hwang.

**Visualization:** Gun Woo Kim.

**Writing – review & editing:** Sung Hoon Cho, Deokbi Hwang, Kyoung Hoon Lim.

**Investigation:** Deokbi Hwang.

**Conceptualization:** Kyoung Hoon Lim.

**Supervision:** Kyoung Hoon Lim.
